# Face-Masking, an Acceptable Protective Measure against COVID-19 in Ugandan High-Risk Groups

**DOI:** 10.4269/ajtmh.20-1174

**Published:** 2020-12-14

**Authors:** Gerald Mboowa, David Musoke, Douglas Bulafu, Dickson Aruhomukama

**Affiliations:** 1 Department of Immunology and Molecular Biology, College of Health Sciences, School of Biomedical Sciences, Makerere University, Kampala, Uganda;; 2 The African Center of Excellence in Bioinformatics and Data Intensive Sciences, The Infectious Diseases Institute, Makerere University, Kampala, Uganda;; 3 Department of Disease Control and Environmental Health, School of Public Health, College of Health Sciences, Makerere University, Kampala, Uganda;; 4 Department of Medical Microbiology, College of Health Sciences, School of Biomedical Sciences, Makerere University, Kampala, Uganda

## Abstract

Face-masking could reduce the risk of COVID-19 transmission. We assessed knowledge, attitudes, perceptions, and practices toward COVID-19 and face-mask use among 644 high-risk individuals in Kampala, Uganda. In data analysis, descriptive, bivariate, and multivariate logistic regression analyses with a 95% CI were considered. Adjusted odds ratios were used to determine the magnitude of associations. *P*-values < 0.05 were considered statistically significant. The majority, 99.7% and 87.3% of the participants, respectively, had heard about COVID-19 and believed that face-masks were protective against COVID-19, whereas 67.9% reported having received information on face-mask use. Food-market vendors and those with no formal education were 0.5 and 0.3 times less likely to have received information about face-mask use than hospital workers and those who had completed secondary school, respectively. Those who had received information on face-mask use were 2.9 and 1.8 times more likely to own face-masks and to perceive them as protective, respectively. Food-market vendors were 3.9 times more likely to reuse their face-masks than hospital workers. Our findings suggest that Ugandan high-risk groups have good knowledge, optimistic attitudes and perceptions, and relatively appropriate practices toward COVID-19.

## INTRODUCTION

As per the third of November 2020, Uganda had 11,767 and 106 COVID-19–confirmed cases and deaths, respectively; of these, 1,943 and 37 COVID-19–confirmed cases and deaths, respectively, were from Kampala district, Uganda’s capital.^1^ COVID-19 is an acute respiratory infectious disease caused by SARS-CoV-2 that spreads mainly through respiratory droplets and secretions.^2,3^ The disease was first reported in Wuhan, Hubei Province of China in December 2019.^4^ COVID-19 transmission can occur directly via contact with symptomatic or presymptomatic or asymptomatic individuals, or indirectly via contact with surfaces in their immediate environment or objects used on or by those infected.^2,5–9^ In specific circumstances and settings particularly where procedures that generate aerosols are performed, airborne transmission of COVID-19 could be possible.^10–12^ The spread of COVID-19 via aerosols even in the absence of aerosol-generating procedures could also be possible.^10–12^ To date, no proven effective treatment options have been reported for the virus; however, the use of hydroxychloroquine and azithromycin has been recommended.^13,14^

To contain viral spread, several countries continue to use non-pharmaceutical public health interventions,^15–17^ including among others border control or closure, partial or complete lockdown, quarantine and testing of incoming travelers and returnees, and mass testing for rapid case detection, contact tracing, and quarantine.^18^ Additional measures; community mitigation strategies including the following among others: mass media-based sensitization and appealing to the masses to do the following: continuously carry out good hygiene practices particularly handwashing, maintain appropriate social distance, limit the numbers attending public gatherings, limit socioeconomic activities except essential services such as security, food markets, and health care; and wear face-masks also continue to be emphasized.^18–20^

These measures have been implemented at different time points and to various degrees in different geographical areas to reduce the risk of community transmission of COVID-19.^18–20^ Noteworthy, several of these measures had been used previously for the control of community transmission of the SARS in 2003, pandemic Influenza A H1N1 in 2009,^3,21,22^ Ebola viral hemorrhagic fever in West Africa in 2014,^23,24^ as well as several viral hemorrhagic fever outbreaks over the years in Uganda.^25^

Wearing of face-masks in public settings, where social distancing measures are difficult to maintain, has been documented as one of the most important prevention measure that can limit the acquisition and spread of COVID-19 by the WHO and the U.S. CDC. In light of this, the WHO and CDC have developed guidelines for the use of the same in these settings.^26,27^

Previously published studies have shown that wearing of face-masks to control infectious disease spread has several advantages that include the following among others: simple operation, strong sustainability, high health benefits, and good health economic benefits.^28–30^ Other previously published studies have also shown that the use of face-masks by the general public is of potentially high value in limiting community transmission of infectious diseases.^3,31–33^ Likewise, the use of face-masks has also been documented to curb viral transmission by asymptomatic individuals, thus limiting the epidemic’s growth rate.^33^ With regard to limiting community spread of COVID-19, community-wide use of face-masks has been encouraged.^34,35^ Face-masks have also been suggested to serve as visible cues of an otherwise yet widely prevalent pathogen, SARS-CoV-2, and as tools that could be used to remind people of the importance of the other infection control measures such as social distancing.^36^ Face-masks are also symbolic; beyond them being tools, they are talismans that could increase healthcare workers’ perceived sense of safety, well-being, and trust in their healthcare settings.^36^

At the time of the study, similar to a few other countries, Uganda was implementing a phased approach of lifting the countrywide lockdown while considering the wearing of face-masks in all public settings mandatory for all.^37^ In light of this, we hypothesized that high knowledge levels about COVID-19 and face-mask use, positive attitudes, and perceptions toward face-mask use as well as good face-mask use practices in Uganda could significantly contribute to breaking the chain of SARS-CoV-2 transmission in healthcare settings and the community via reducing the infectiousness of the subclinical virus shedders while also offering some protection to the susceptible populations.

Hence, we aimed to provide evidence on healthcare and community-level perspectives on the use of face-masks in preventing COVID-19 acquisition and spread through assessing the knowledge, attitudes, perceptions, and practices toward their use. This is because literature remains scarce regarding the use of face-masks in Uganda. We hoped that our findings could be used by decision-makers to guide their recommendations with regard to the use of face-masks by the population, including healthy, presymptomatic, and asymptomatic individuals in healthcare settings and the community to prevent healthcare settings and community acquisition and spread of COVID-19.

## MATERIALS AND METHODS

### Study sites and settings.

This study was carried out in Kampala, the capital city of Uganda. Kampala is divided into five divisions, namely Central, Kawempe, Makindye, Nakawa, and Rubaga. Study sites were purposively selected to represent these five divisions and included 1) food markets, namely i) Owino market located in downtown Kampala, ii) Nakasero market located at the foot of Nakasero hill, iii) Bugolobi market located along Old Portbell road, iv) Nakawa market located along Kampala–Jinja highway, v) Kalerwe market located on the Kampala northern bypass along Gayaza road, vii) Kasubi market located along Kampala–Hoima road, and viii) Wandegeya market located in front of the four-way junction north and northeast of Makerere University, east and north of Mulago National Referral Hospital, and south and southeast of Nakasero hill^38^; 2) police stations, namely central, Old Kampala, Katwe, Mulago, Kanyanya, and Wandegeya; and 3) Mulago National Referral Hospital, the largest public hospital in Uganda located on Mulago hill in the northern part of Kampala.

### Study design.

This study was a cross-sectional study, and was part of a larger study titled: Assessing knowledge, attitudes, perceptions, and skills toward the use of face-masks: a community-level perspective (MASKUG-2020), that aimed to assess knowledge, attitudes, perceptions, and practices toward the use of face-masks by high-risk groups in Kampala district, Uganda.

### Study population, period, and high-risk group definition.

The study population comprised high-risk groups, namely 1) food-market vendors that included food store owners and sellers of fruits and vegetables, 2) police officers mainly traffic officers and curfew enforcers, and 3) healthcare workers mainly nurses and medical doctors. All these individuals had been allowed to continue their businesses during the entire countrywide lockdown ordered by the Ugandan government; this was because they were considered as essential service providers.^39^ However, this study was conducted in July 2020, during the time when the entire country lockdown had been eased.^40^

This study defined individuals in high-risk groups as those who were working in the selected sites, who by virtue of their occupations in these sites would have inevitably continuously interacted with multiple different people on a day-to-day basis. This meant that they were at a higher risk of contracting and/or transmitting COVID-19. The choice to define these individuals as high-risk groups was based on several studies that demonstrated that crowding influences the overall load of infectious agents, including respiratory viruses.^41,42^

### Sample size and sampling.

This study’s sample size constituted 659 study participants. The sample size was calculated using the Kish Leslie formula (1995) for cross-sectional studies, giving a sample size of 384. Because most of the targeted participants were working in shifts, we considered a nonresponse rate of 30%, and a design effect of 1.2,^43^ giving us a sample size of 659 study participants. At each of the sites, multistage sampling was performed based on the average number of participants present to ensure equal representation of all the sites. Several clusters of 20–25 participants were selected from each site using the probability proportion to size sampling, which ensured that all individuals in the target populations had an equal chance of being selected. Three to four busy days of the week were purposively selected to visit each of the sites (Table 1).

**Table 1:** Sample sizes and sampling

Study sites	Frequency (*N* = 644)	Percentage
Markets (*n* = 381)		
Owino	70	10.9
Kasubi	69	10.7
Kalerwe	59	9.2
Nakawa	40	6.2
Nakasero	59	9.2
Bugolobi	40	6.2
Wandegeya	44	6.8
Police stations (*n* = 182)		
Katwe	14	2.2
Central	57	8.9
Mulago	11	1.7
Kanyanya	28	4.4
Wandegeya	29	4.5
Old Kampala	43	6.7
Hospital (*n* = 81)		
Mulago National Referral Hospital	81	12.6

### Questionnaire design.

A semi-structured questionnaire based on the Occupational Safety and Health Administration (OSHA) Respiratory Protection Program standard requirements (OSHA, 2017) and the Guyanan Ministry of Health in partnership with UNICEF-Knowledge, Attitudes and Practices (KAP) survey on COVID-19 response^44^ was developed and used in the data collection. One occupational/environmental health and safety expert, a statistician, and three healthcare workers (i.e., one doctor and two nurses) assessed the validity of the questionnaire. The reliability of the questionnaire was checked by Cronbach’s alpha (α = 0.860, 0.899, and 0.870, respectively, for knowledge, attitudes and perceptions, and practices dimensions). The questionnaire consisted of five components including demographics, knowledge (12 items), attitudes, perceptions, and practices (10 items). Knowledge items were categorized as yes (score 1) and no (score 0). Attitudes, perceptions, and practices items were scored using a Likert scale, which ranged from one (very fearful) to four (optimistic) and one (strongly agree) to four (strongly disagree). Other attitudes, perceptions, and practice items were categorized as yes (score 1) and no (score 0). All negatively worded responses were scored reversely. In addition, the study questionnaire was evaluated for face and internal validity by the investigators. To enhance data quality, all research assistants (RAs) were trained and supervised, and the questionnaire was pretested.

### Data collection, validation, and analysis.

Data were collected by the trained RAs using the developed semi-structured questionnaires. In brief, the data were entered using mobile android and iPhone Operating System (iOS) phones and tablets. These had been loaded with the Open Data Kit application (ODK, University of Washington, Seattle, WA), and the data were synchronized onto a remote server daily. Data collection using mobile android and iOS phones and tablets allowed for real-time data capture and entry, minimized errors at entry, and eased data cleaning. To ensure that the data were secure, only the principal investigators had the security key to the ODK server, where the data were being sent during data collection. Validation of the collected data was performed by checking a significant percentage (20–30%) of the same by field supervisors and the principal investigators. The collected data were cleaned using Microsoft Excel 2016 (Microsoft Corporation, Redmond, WA) and analyzed using STATA 14.0 statistical software (StataCorp, College Station, TX). Descriptive analyses such as frequencies, proportions, and means (where appropriate) were performed for demographic characteristics, as well as for knowledge, attitudes, perceptions, and practices toward face-mask use. To assess the association between the outcome variables (knowledge on right procedure of wearing face-masks, receipt of information on the use of face-masks, face-mask ownership, and use face-mask reuse, attitudes, perceptions, and practices) and each explanatory variable, we considered a binary logistic regression which provided crude odds ratios (OR) and their corresponding 95% CIs . Variables with *P* < 0.05 were all added into the multivariate logistic regression to ascertain significant variables for each outcome. The statistical significance levels were two-sided at *P* < 0.05.

### Ethics approval and consent to participate.

Ethical approvals were obtained from the 1) School of Biomedical Sciences-Research and Ethics Committee, College of Health Sciences, Makerere University (approval number: SBS-793), Mulago National Referral Hospital-Research and Ethics Committee (approval number: MHREC-1887), and Uganda National Council for Science and Technology (approval number: SS489ES). Administrative clearance was also sought and obtained from the directorate of Research, Planning, and Development of the Uganda Police Force, and incharges of the different wards/units of Mulago National Referral Hospital, the respective police stations, and food markets. Written informed consent/assent was obtained from each of the study participants before collection of the questionnaire data.

## RESULTS

### Social demographics.

A total of 644 participants completed the survey questionnaire, with a response rate of 98%. The average age of the participants was 35.1 years (SD: 11.0, range 14–71), less than half, 40.2% were within 24–33 years of age, and 52.8% were male.

The majority of the participants (82.4%) were Christians, more than half, 59.2% worked in food markets, whereas 51.2% walked to their places of work. The majority (87.2%) of the participants had attended primary school and higher (Table 2).

**Table 2:** Social demographics of study participants

Variable	Frequency (*N* = 644)	Percentage
Age-group (years)		
14–23	71	11.0
24–33	259	40.2
34–43	167	25.9
44–53	104	16.2
54–63	34	5.3
64–73	9	1.4
Gender		
Female	304	47.2
Male	340	52.8
Education level		
Complete secondary	127	19.7
Complete primary	65	10.1
Incomplete primary	57	8.9
Incomplete secondary	191	29.7
No formal education	26	4.0
Technical/vocational	34	5.3
University/tertiary	144	22.4
Religion		
Christian*	531	82.4
Moslem	110	17.1
Other religions†	3	0.5
Commonly used mode of transport		
*Boda boda*‡ (public)	10	1.6
Cycling	16	2.5
Motor bike (private)	35	5.4
Taxi	203	31.5
Private car	50	7.8
Walking	330	51.2
Site		
Hospital	81	12.6
Police stations	182	28.3
Food markets	381	59.2

* Christian included: Catholics, 248 (38.5%), Seventh-day adventists, 11 (1.7%), Pentecostal/Born again, 78 (12.1%) and Anglican, 194 (30.1%).

† Other religions/beliefs included; traditionalists and no religion.

‡ Boda boda is a commercial motorcycle.

### Knowledge about COVID-19 and use of face-masks.

Nearly all (99.7%) participants reported having heard about COVID-19, whereas 98.6% of the participants reported that they had heard and/or seen messages about the disease. The commonly heard and/or seen message reported were handwashing (36.2%), social distancing (21.3%), and wearing of face-masks as a protective measure against COVID-19 (21.4%). The majority of the participants (80.6%) reported having heard and/or seen the messages on local television stations. Other sources of information about COVID-19 reported by the participants included local radio stations (64.3%); family and friends (14.7%); local newspapers (15.6%); social media platforms, for example, Twitter and Facebook among others (29.5%); and other internet platforms, for example, organizational websites among others (5.8%).

The majority (67.9%) of the participants reported having received information on how to use face-masks. A large proportion (80.8%) of those who had received the information received it from local television stations. Other reported sources of the information on how to use face-masks included local leaders or community health workers (CHWs) (26.1%), social media platforms (8.2%), and local radio stations (16.5%). The majority (77.0%) of the participants also reported that they knew the right procedure or steps of wearing face-masks. When asked about face-mask ownership and use, the majority of the participants (67.8%) reported owning and using locally made, nonmedical face-masks, mostly made from single-layered (35.3%) or double-layered (27.7%) cotton fabric (mostly “kitenge,” a local fabric printed in various colors and designs).

### Factors associated with knowledge on the right procedure of wearing face-masks and receiving information on the use of face-masks.

Bivariate analysis showed that age and receipt of information on face-mask use among the participants were the factors associated with knowledge on the right procedure of wearing face-masks. Individuals aged between 34 and 43 years (OR: 1.87; 95% CI: 1.00–3.50) were 1.87 times more likely to know the right procedure of wearing face-masks than those aged between 14 and 23 years. Study participants who had received information on the use of face-masks (OR: 6.96; 95% CI: 4.66–10.40) were 6.96 times more likely to be know the right procedure of wearing face-masks than those who had never received information on the same.

The bivariate analysis also showed that age, gender, education level, and the site of work were the factors associated with receiving information on the use of face-masks. Individuals aged 24–33 years (OR: 2.05; 95% CI: 1.20–3.51) were 2.05 times more likely to receive information on the use of face-masks, those aged 34–43 years (OR: 1.92; 95% CI: 1.09–3.40) were 1.92 times more likely to receive information on the use of face-masks, and those aged 44–53 years (OR: 2.14; 95% CI: 1.14–4.02) were 2.14 times more likely to receive information on use of face-masks than those aged 14–23 years.

Males (OR: 0.62; 95% CI: 0.44–0.86) were 38% less likely to receive information on the use of face-masks than females. Those with no formal education (OR: 0.28; 95% CI: 0.12–0.66) were 72% less likely to have received the information than those who had completed secondary education. Participants who worked in the food markets (OR: 0.36; 95% CI: 0.19–0.66) were 64% less likely to have received the information, whereas those who worked at police stations (OR: 0.51; 95% CI: 0.26–0.98) were 49% less likely to have received the information than those who worked in the hospital.

After adjusting for confounding, only those who had received information on the use of face-masks (Adjusted odds ratios [AOR]: 6.72; 95% CI: 4.47–10.08) were 6.72 times more likely to know the correct procedure of wearing face-masks than those that did not receive the information. Furthermore, individuals aged 24–33 years (AOR: 1.9; 95% CI: 1.08–3.35), 44–53 years (AOR: 2.12; 95% CI: 1.09–4.14), and 54–63 years (AOR: 3.39; 95% CI: 1.29–8.89) were more likely to have received information on how to use face-masks than those aged 14–23 years. Males (AOR: 0.58; 95% CI: 0.40–0.83) were less likely to have received information on the use of face-masks. Those with no formal education (AOR: 0.25; 95% CI: 0.09–0.63), were less likely to have received information on the use of face-masks than those who completed secondary education. Last, those who worked in food markets (AOR: 0.47; 95% CI: 0.24–0.93) were also less likely to have received the information as than those who worked in hospital (Table 3).

**Table 3:** Factors associated with knowing the right procedure and receipt of information on the use of face-masks

Demographics	Know correct procedure of wearing a mask				Received information on face-mask use			
	Unadjusted OR (95% CI)	*P*-value	Adjusted OR (95% CI)	*P*-value	Unadjusted OR (95% CI)	*P*-value	Adjusted OR (95% CI)	*P*-values
Age-group (years)								
14–23	–	–	–	–	–	–	–	–
24–33	1.78 (1.00–3.17)	0.052	1.36 (0.72–2.57)	0.341	2.05 (1.20–3.51)	0.009	1.90 (1,08–3.35)	0.025
34–43	1.87 (1.00–3.50)	0.048	1.50 (0.76–2.96)	0.245	1.92 (1.09–3.40)	0.025	1.68 (0.28–1.19)	0.091
44–53	1.44 (0.74–2.80)	0.286	1.04 (0.50–2.16)	0.923	2.14 (1.14–4.02)	0.018	2.12 (1.09–4.14)	0.027
54–63	2.77 (0.95–8.11)	0.061	1.95 (0.62–6.17)	0.254	2.82 (1.12–7.08)	0.027	3.39 (1.29–8.89)	0.013
64–73	0.39 (0.94–1.56)	0.181	0.60 (0.13–2.75)	0.514	0.25 (0.05–1.28)	0.096	0.28 (0.05–1.52)	0.139
Gender								
Female	–	–	–	–	–	–	–	–
Male	0.730 (0.5–1.05)	0.097			0.62 (0.44–0.86)	0.005	0.58 (0.40–0.83)	0.003
Education level								
Complete secondary	–	–	–	–	–	–	–	–
Complete primary	1.52 (0.70–3.26)	0.286	–	–	1.16 (0.60–2.24)	0.665	1.12 (0.55–2.28)	0.761
Incomplete primary	1.05 (0.50–2.20)	0.904	–	–	0.57 (0.30–1.08)	0.085	0.57 (0.28–1.19)	0.133
Incomplete secondary	0.71 (0.42–1.18)	0.189	–	–	0.86 (0.53–1.39)	0.536	0.83 (0.49–1.40)	0.483
No formal education	0.70 (0.28–1.76)	0.444	–	–	0.28 (0.12–0.66)	0.004	0.25 (0.09–0.63)	0.003
Technical/vocational	1.19 (0.47–3.01)	0.709	–	–	0.93 (0.41–2.09)	0.854	0.93 (0.41–2.14)	0.873
University/tertiary	1.81 (0.98–3.36)	0.059	–	–	1.49 (0.87–2.56)	0.148	1.21 (0.69–2.12)	0.515
Religion								
Catholic	0.37 (0.05–2.94)	0.346	–	–	0.48 (0.10–2.29)	0.361	–	–
Moslem	0.21 (0.03–1.67)	0.139	–	–	0.36 (0.07–1.75)	0.205	–	–
SDA	–	–	–	–	–	–	–	–
Pentecostal/born again	0.50 (0.06–4.25)	0.526	–	–	0.80 (0.16–4.04)	0.785	–	–
Protestant	0.33 (0.04–2.66)	0.298	–	–	0.42 (0.09–2.00)	0.278	–	–
Other religions	1		–	–	0.44 (0.03–7.67)	0.577	–	–
Site category								
Hospital	–	–	–	–	–	–	–	–
Police station	0.86 (0.44–1.67)	0.660	–	–	0.51 (0.26–0.98)	0.044	0.56 (0.28–1.12)	0.102
Market	0.68 (0.37–1.26)	0.220	–	–	0.36 (0.19–0.66)	0.001	0.47 (0.24–0.93)	0.029
Received information on face-mask use								
No	–	–	–	–	–	–	–	–
Yes	6.96 (4.66–10.40)	0.000	6.72 (4.47–10.08)	< 0.001	–	–	–	–

OR = odds ratio; SDA = Seventh-day adventists.

### Attitudes and perceptions on COVID-19 and use of face-masks.

The majority (82.5%) of the participants reported that they feared (41.5%) and were very fearful (41.0%) of COVID-19. Likewise, the majority (91.6%) of the participants reported that they agreed (52.2%) and strongly agreed (46.1%) that acquiring COVID-19 is serious.

Furthermore, the majority (87.3%) of the participants agreed (52.2%) and strongly agreed (35.1%) that face-masks are a good protective measure against COVID-19. With regard to whether or not participants would indefinitely wear face-masks if the COVID-19 threat persisted, the majority (68.6%) reported that they would. Others (31.4%) reported that they would not, as the majority (81.7%) thought it is would be an inconvenience. The majority (88.2%) of the participants also reported that they would readily wear face-masks if everyone in their communities was wearing one.

A large proportion (82.4%) of the participants reported that they would easily wear face-masks if there were banners and posters available to remind them do so. Others (81.6%) thought that the other ways that could remind them about wearing face-masks would be local television and radio stations. More than half (71.1%) of the participants thought the government’s response to COVID-19 was adequate.

### Factors associated with attitudes and perceptions toward COVID-19 and face-mask use.

Bivariate analysis showed that only receiving information on face-mask use was associated with whether one would be comfortable wearing a face-mask indefinitely if COVID-19 persisted. Those who received information on the use of face-masks (OR: 1.58; 95% CI: 1.11–2.23) were 1.58 times more likely to be comfortable wearing them indefinitely if COVID-19 persisted.

The bivariate analysis also showed that age, gender, education level, and receipt of information on face-mask use were the factors associated with people’s perception on whether a mask is a good protective measure against COVID-19. Participants aged 64 years and older (OR: 0.18; 95% CI: 0.04–0.80) were 89% less likely to perceive the use of face-masks as a good protective measure against COVID-19 than those younger than 64 years. Male participants (OR: 0.61; 95% CI: 0.38–0.97) were 39% less likely to perceive the use of face-masks as a good protective measure against COVID-19 than females. Those who completed primary school (OR: 3.64; 95% CI: 1.03–12.78) were three times more likely to perceive the use of face-masks as a good protective measure against COVID-19 than those who had completed secondary school.

After adjusting for confounders, those aged 64 years and older (AOR: 0.17; 95% CI: 0.03–0.82) (83%) were less likely to perceive the use of face-masks as good protective measures against COVID-19 than those younger than 64 years. Those who received information on the use of face-masks (AOR: 1.83; 95% CI: 1.11–3.02) were more likely to perceive the use of face-masks as good protective measures against COVID-19 than those who had never received the same information (Table 4).

**Table 4:** Factors associated with attitudes and perceptions toward COVID-19 and face-mask use

Demographics	Comfortably wear face-mask indefinitely if COVID-19, persists				Mask is a good protective measure			
	Unadjusted OR (95% CI)	*P*-value	Adjusted OR (95% CI)	*P*-value	Unadjusted OR (95% CI)	*P*-value	Adjusted OR (95% CI)	*P*-value
Age-group (years)								
14–23	–	–	–	–	–	–	–	–
24–33	1.14 (0.65–2.00)	0.640	–	–	0.84 (0.39–1.84)	0.670	0.84 (0.37–1.90)	0.667
34–43	0.82 (0.46–1.47)	0.509	–	–	1.28 (0.54–3.03)	0.573	1.32 (0.54–3.23)	0.541
44–53	1.90 (0.96–3.76)	0.064	–	–	1.23 (0.48–3.13)	0.669	1.13 (0.43–2.97)	0.801
54–63	1.23 (0.51–2.97)	0.653	–	–	1.50 (0.38–5.94)	0.564	1.26 (0.304–5.17)	0.753
64–73	1.79 (0.34–9.27)	0.489	–	–	0.18 (0.04–0.80)	0.025	0.17 (0.03–0.82)	0.027
Gender								
Female	–	–	–	–	–	–	–	–
Male	0.83 (0.60–1.16)	0.280	–	–	0.61 (0.38–0.97)	0.041	0.66 (0.40–1.09)	0.104
Education level								
Complete secondary	–	–	–	–	–	–	–	–
Complete primary	1.83 (0.92–3.62)	0.083	–	–	3.64 (1.03–12.78)	0.044	3.40 (0.95–12.23)	0.061
Incomplete primary	1.69 (0.83–3.41)	0.146	–	–	1.07 (0.44–2.63)	0.870	1.32 (0.50–3.49)	0.573
Incomplete secondary	1.29 (0.80–2.08)	0.296	–	–	1.50 (0.77–2.95)	0.234	1.52 (0.76–3.09)	0.235
No formal education	1.48 (0.58–3.81)	0.406	–	–	0.74 (0.25–2.20)	0.586	0.86 (0.27–2.74)	0.809
Technical/vocational	1.31 (0.58–3.00)	0.512	–	–	0.68 (0.26–1.78)	0.430	0.73 (0.27–1.93)	0.522
University/tertiary	0.91 (0.56–1.50)	0.724	–	–	1.09 (0.55–2.15)	0.802	0.97 (0.49–1.94)	0.937
Religion								
Catholic	1.64 (0.47–5.80)	0.440	–	–	2.40 (0.49–11.85)	0.282	–	–
Moslem	0.92 (0.26–3.35)	0.906	–	–	0.76 (0.15–3.73)	0.731	–	–
SDA	–	–	–	–	–	–	–	–
Pentecostal/born again	1.45 (0.39–5.47)	0.579	–	–	1.51 (0.28–8.03)	0.628	–	–
Protestant	1.05 (0.30–3.75)	0.929	–	–	1.65 (0.34–8.12)	0.537	–	–
Other religions	0.29 (0.02–4.24)	0.363	–	–	0.44 (0.02–7.67)	0.577	–	–
Site category								
Hospital	–	–	–	–	–	–	–	–
Police station	0.57 (0.31–1.03)	0.061	–	–	0.69 (0.28–1.68)	0.412	–	–
Market	0.67 (0.39–1.18)	0.167	–	–	0.59 (0.26–1.34)	0.205	–	–
Received information on face-mask use								
No	1.58 (1.11–2.23)	0.011	–	–	–	–	–	–
Yes	–	–	–	–	2.01 (1.26–3.21)	0.004	1.83 (1.11–3.02)	0.018

OR = odds ratio.

### Practices toward the use of face-masks.

Almost all (93.3%) the participants had done something to protect themselves and their families from COVID-19. The majority (81.4%) had practiced handwashing with soap and water for at least 20 seconds, whereas more than half (70.6%) had worn or used face-masks. The majority (82.1%) of the participants reported that they had reused their face-masks whether or not they were reusable. In addition, the majority (41.4%) of those who reused their face-masks reported that they had done so for 1 week or less, whereas a significant number (23.1%) of participants reported that they had reused their face-masks for more than 1 month.

### Factors affecting the practices on the use of face-masks.

Bivariate analysis showed that age, site of work, and receipt of information on the use of face-masks were the factors associated with ownership and use of face-masks, whereas education status and site of work were the factors associated to reuse of face-masks. Participants aged 24–33 years (OR: 2.78; 95% CI: 1.23–6.31) and those within 34–43 (OR: 2.60; 95% CI: 1.07–6.31) were more likely to own face-masks than those aged 14–23 years. Study participants who worked in the food markets (OR: 0.34; 95% CI: 0.15–0.78) were 66% less likely to own face-masks than those who worked in the hospital. Those who had received information on the use of face-masks (OR: 3.44; 95% CI: 1.87–6.32) were 3.44 times more likely to own face-masks than those who never received information on the same.

Participants who had completed primary school (OR: 5.36; 95% CI: 1.55–18.49) were 5.36 times more likely to reuse their masks than those who had completed secondary school, and those who had not completed primary school (OR: 3.30; 95% CI: 1.09–10.00) were three times more likely to reuse their face-masks than those who had completed secondary school. Participants who worked in food markets (OR: 4.61; 95% CI: 2.47–8.59) were 4.61 times more likely to reuse their face masks than those who worked in the hospital.

At multivariate analysis, participants who worked in food markets (AOR: 0.38; 95% CI: 0.16–0.88) were 62% less likely to own face-masks than their counterparts who worked in the hospital. Those who had received the information on the use of face-masks (AOR: 2.85; 95% CI: 1.53–5.32) were 2.85 times more likely to own face-masks than those who had not received information on the use of face-masks. Furthermore, those who worked in the food markets (AOR: 3.92; 95% CI: 1.97–7.82) were 3.92 times more likely to reuse their face-masks than those who worked in the hospital (Table 5).

**Table 5:** Factors affecting face-mask use practices

Demographics	Own face mask				Reuse face mask			
	Unadjusted OR (95% CI)	*P*-value	Adjusted OR (95% CI)	*P*-value	Unadjusted OR (95% CI)	*P*-value	Adjusted OR (95% CI)	*P*-value
Age-group (years)								
14–23	–	–	–	–	–	–	–	–
24–33	2.78 (1.23–6.31)	0.014	2.05 (0.88–4.76)	0.097	0.74 (0.33–1.66)	0.462	–	–
34–43	2.60 (1.07–6.31)	0.035	1.61 (0.64–4.09)	0.311	0.57 (0.24–1.33)	0.193	–	–
44–53	2.54 (0.93–6.91)	0.068	1.63 (0.58–4.59)	0.352	0.68 (0.27–1.67)	0.394	–	–
54–63	6.05 (0.75–48.95)	0.092	4.45 (0.54–36.92)	0.167	1.12 (0.31–4.02)	0.868	–	–
64–73	1.47 (0.17–12.92)	0.730	2.21 (0.24–20.17)	0.483	0.46 (0.08–2.70)	0.391	–	–
Gender								
Female	–	–	–	–	–	–	–	–
Male	0.82 (0.45–1.49)	0.508	–	–	1.15 (0.76–1.75)	0.508	–	–
Education level								
Complete secondary	–	–	–	–	–	–	–	–
Complete primary	0.49 (0.15–1.58)	0.230	–	–	5.36 (1.55–18.49)	0.008	2.11 (0.57–7.80)	0.263
Incomplete primary	0.35 (0.11–1.11)	0.074	–	–	3.30 (1.09–10.00)	0.034	1.14 (0.35–3.78)	0.826
Incomplete secondary	0.49 (0.17–1.16)	0.097	–	–	1.48 (0.82–2.66)	0.194	0.94 (0.49–1.81)	0.861
No formal education	0.60 (0.11–3.13)	0.540	–	–	2.01 (0.56–7.25)	0.286	0.73 (0.19–2.90)	0.659
Technical/vocational	1.64 (0.19–14.07)	0.654	–	–	1.29 (0.48–3.45)	0.609	1.04 (0.37–2.94)	0.932
University/tertiary	1.14 (0.36–3.63)	0.824	–	–	0.81 (0.46–1.44)	0.480	0.92 (0.49–1.72)	0.802
Religion								
Catholic	1.49 (0.73–3.07)	0.277	–	–	1.28 (0.78–2.09)	0.325	–	–
Moslem	0.86 (0.39–1.92)	0.720	–	–	1.81 (0.91–3.59)	0.090	–	–
SDA	–	–	–	–	1	–	–	–
Pentecostal/born again	1.78 (0.58–5.46)	0.316	–	–	1.10 (0.55–2.10)	0.832	–	–
Protestant	1	–	–	–	1	–	–	–
Other religions	1	–	–	–	1	–	–	–
Site category			–	–	1	–	–	–
Hospital	–	–	–	–	0.82 (0.46–1.46)	0.503	0.78	0.424
Police station	1	–	–	–	4.61 (2.47–8.59)	< 0.001	3.92 (1.97–7.82)	< 0.001
Market	0.34 (0.15–0.78)	0.010	0.38 (0.16–0.88)	0.025	–	–	–	–
Received information on face-mask use								
No	1	–	–	–	–	–	–	–
Yes	3.44 (1.87–6.32)	< 0.001	2.85 (1.53–5.32)	0.001	–	–	–	–

OR = odds ratio.

## DISCUSSION

To the best of our knowledge, this is the first study assessing the knowledge, attitudes, perceptions, and practices toward COVID-19 and the use of face-masks among Ugandan high-risk groups. In this study, we analyzed knowledge on the right procedure of face-mask use, receipt of information on the use of face-masks, and face-mask ownership and use as well as their associated factors. These findings could be useful for public health policy-makers, health workers, and other stakeholders to improve the uptake of face-masks as a key intervention in the prevention of COVID-19, for example, through health education among key populations.

In this study, most of the participants reported having heard about COVID-19, an indication that they were knowledgeable about the disease. Most of the participants held non-optimistic attitudes and perceptions toward COVID-19. Indeed, many participants reported that they were fearful about the disease and also agreed that contracting the virus was serious. In light of this, the practices of the participants were very cautious as nearly all reported having done something to protect themselves and their families from COVID-19. Not only could have these practices been primarily attributed to their fear of contracting COVID-19, but they could also have been due to the strict prevention and control measures that had been implemented by the Ugandan government such as banning of all public gatherings among others. Also, the practices could have also been the result of the target populations’ high level of knowledge regarding the seriousness of contracting COVID-19.

The participants however believed that the government’s response to COVID-19 had been adequate. This could be attributed to the actions the government had undertaken in the early stages of the pandemic that included suspension of all public gatherings, closure of all schools, and suspension of public transport among others.^45–47^ These actions could have positively affected the perceptions and practices toward COVID-19.

Unlike a related study carried out in China that reported unexpected high COVID-19 knowledge levels among the population during the rapid rise of the COVID-19 outbreak,^48^ the finding in this study where most of the participants had reported being knowledgeable about COVID-19 was expected. This is because this study was conducted during the time Uganda’s COVID-19 infections had entered stage three (i.e., community transmission) as had been declared by her Ministry of Health in a press release in early June 2020.^49^ However, the finding could also be attributed to the efforts that had been pursued by the Ugandan government specifically her Ministry of Health to educate the population about the disease, across several fora such as local television and radio stations. This finding is also similar to that in studies elsewhere that have reported high levels of COVID-19 knowledge in groups similar to this study’s target populations or rather the high-risk groups.^50–53^ Improved knowledge on infectious diseases has been shown to avert negative attitudes while promoting positive preventive practices.^53^ We also believe that the aforementioned finding could be due to the participants’ attitudes and perceptions toward COVID-19. Indeed, most of the participants reported that they feared COVID-19. Because of the threat of the pandemic and the overwhelming news reports on this public health emergency, these populations could have heard of COVID-19 from various channels of information. These sources of information included local newspapers, television and radio stations, social media, and other internet platforms, notably the organizational websites, such as Twitter and Facebook accounts of the Uganda’s Ministry of Health and Makerere University, Uganda’s largest and oldest institution of higher learning.^54,55^

Uganda has in the past experienced several viral disease outbreaks during which it has learned invaluable lessons on how best to deal with these epidemics. Indeed, most of the population have developed belief in their government’s ability to respond to these diseases, as these responses have been refined over time.^25,56–58^ In the case of this study, the belief that the Ugandan government’s response was adequate could be related to the manner in which the country handled previous viral diseases outbreaks; hence, belief already instilled in the Ugandan population but also the unprecedented COVID-19 control measures such as the lockdown of the entire country, willingness to heed to the call sent across by the Ugandan government for concerted efforts from across the country particularly the business community, religious and cultural institutions to comply with the directives provided by the Ugandan Ministry of Health and cease conducting business, indefinitely suspend religious and cultural gatherings while encouraging their followers to observe all the guidelines provided to prevent the transmission of COVID-19,^59^ could have also increased the confidence of the Ugandans, as it demonstrated the belief that the different stakeholders had in the government’s capability to handle the situation, and high knowledge levels about COVID-19 among the target groups could also explain this phenomenon, as increase in knowledge could have been attributed mostly to the efforts of the Ugandan government.

Fortunately, the present study like other related studies^48,52,53,60,61^ showed that despite the use of face-masks not being a norm in the Ugandan society and the shortage of supply of face-masks due to their high demand as reported elsewhere,^50,62^ most of the participants owned and had used face-masks as a protective measure against COVID-19. The participants also reported that they had received information on the use of face-masks via various channels: local leaders and CHWs, local television and radio stations, as well as social media and other Internet platforms, and believed that they knew the right procedures of how to use face-masks. This finding is consistent with those of other studies that have showed that these platforms constitute the major sources of information about COVID-19.^52,53,63^ In addition, the transition from television and radios to social media and other Internet platforms continues at an unprecedented rate in Uganda.^64^ Indeed, the use of smart phones continues to increase across the country, Internet connectivity is currently progressing from a luxury for the rich to a felt need for the middle class, and Internet cafes are still flourishing throughout the capital city Kampala with lower prices.^64^ These developments in the country could explain the increasing use of social media and other Internet platforms as sources of information on COVID-19 for the population.^64^ Also, over time, local leaders and CHWs have continued to play a critical role in information dissemination particularly during disease outbreaks in Uganda.^65^ Similar to a recommendation of another study in the same setting,^53^ this finding underscores the need to frequently use such media to disseminate COVID-19–related information. In addition, this study underscores the need to use local leaders and CHWs in the dissemination of COVID-19–related information, in addition to the different media platforms.

In this study, knowledge about the right procedure of wearing face-masks was related to receipt of information toward the use of face-masks which was related to age, gender, education levels, and site of work, whereas the decreased likelihood of receiving information on the use of face-masks was related to the young (24–33 years of age), males, having no formal education, and working in food markets. Previous studies regarding age and gender patterns of risk-taking behaviors^66–68^ have showed that men and late adolescents or the young are more likely to engage in risk-taking behaviors. These findings could explain the less likelihood of the males and late adolescents or the young receiving information on face-masks use as well as perceiving the use of face-masks as a protective measure against COVID-19 in this study. However, these findings could also be explained by the normally held beliefs by men with regard to masculinity that have been recurrently blamed for health attitudes that could negatively influence men’s health, lower their life expectancy, and increase their morbidity rates as reported in a previous study that analyzed associations between masculine norms and healthcare utilization in highly religious, heterosexual men.^69^

Our study showed a high level of COVID-19 awareness as well as a high level of knowledge about the right procedure of wearing face-masks among the participants. This finding signifies a positive predictor in curtailing the COVID-19 pandemic within high-risk groups in Uganda. Strictly speaking, our study findings can only be generalized to Ugandan populations of a relatively high socioeconomic status. Considering that educational attainment and occupation are often used as proxy measures of socioeconomic status,^48^ these findings excluded the underprivileged. The likely diminished understanding of the English language and the reduced likelihood of owning either a television set, radio, or mobile phone or even accessing the Internet and online information resources in these particular populations underscores the need to pursue research on knowledge, attitudes, perceptions, and practices toward COVID-19 in these populations in Uganda, identify other platforms/means of disseminating knowledge with regard to COVID-19 and practices thereof. Efforts to use local leaders and CHWs as well as the dissemination of knowledge pertaining COVID-19 in various local languages could also be pursued.

Unlike the findings of related studies where ownership and use of face-masks were less common,^50,53^ most of this study’s participants owned and used face-masks, and believed that the use of the face-masks would protect them from contracting COVID-19. However, this study’s findings are similar to those of other studies.^52,70,71^ Age and receipt of information on the use of face-masks were the factors that were associated with people’s attitudes and perceptions on whether face-masks were a good protective measure against COVID-19. Participants also reported that they would wear face-masks indefinitely in case the COVID-19 threat persisted, and suggested that with constant reminders (especially via banners and posters, television, and radio reminders) and watching others in their settings/communities wearing them, they would continue wearing their face-masks. This finding is consistent with the perspective that face-masks are beyond simply pieces of fabric but rather symbols that serve as constant reminders, and that indicate the presence of a threat(s).^36^ This finding suggests that face-masks could be leveraged as symbols that could gradually impact attitudes, perceptions, and practices toward COVID-19 in these populations while offering protection against acquiring the virus.

Despite the low certainty evidence as alluded in a number of studies and perspectives,^36,72^ regarding the protection offered by face-masks in the prevention of COVID-19, our findings on ownership and the use of face-masks by the participants were expected and could be explained by their fear of COVID-19 and the perceived seriousness of contracting the virus. This explanation has also been expounded in the perspective,^36^ in which expanded masking protocols’ greatest contribution was noted as their role in reducing the transmission of nervousness, over and above whatever role they could play in reducing COVID-19 transmission. The findings on the perceived role of face-masks in preventing the spread of COVID-19 underscore the need to pursue quality, cost-effective research including randomized trials in multiple settings to examine research gaps related to aerosol-generating procedures and airborne transmission of SARS-CoV-2, as face-mask use appears to be an acceptable prevention measure to many.

In the absence of research affirming that face-masks do not offer protection against COVID-19, this study’s findings underscore the need for all countries to critically consider the opinions of available studies that have evaluated presymptomatic and asymptomatic transmission of SARS-CoV-2 and a growing compilation of observational evidence on the use of face-masks by the general public conducted in several countries during the COVID-19 pandemic. In so doing, these countries should adopt the current guidelines provided by the WHO and CDC with regard to the use of face-masks in healthcare and community settings to prevent the infected wearer transmitting the infection to others, offer protection to the health wearer against infection, abate circumstances where there could be high risk of exposure to SARS-CoV-2 due to intensity of transmission and epidemiology in the population coupled with limited or no capacity to implement other containment measures, for example, contact tracing, testing and isolation, and care of suspected and confirmed cases, also depending on occupation: individuals working in close contact with the public offer protection in settings with high population density and settings where individuals are unable to keep a physical distance, particularly those where the risks are greater to ensure a comprehensive approach toward preventing the transmission of COVID-19.^27^

The finding where older participants believed that face-masks were not a good protective measure against COVID-19 may be attributed to their inadequate knowledge about COVID-19, specifically the use of face-masks as a preventive measure against the disease. This is consistent with another study^73^ that reported greater difficulties in accessing novel information, higher likelihood of encountering financial or resource barriers to implement preventive measures among old people, as well as poor neighborhoods and communities.

Unfortunately, some of the participants in this study reported that they could not wear face-masks indefinitely if the COVID-19 threat was to persist, as they found them an inconvenience. This finding could be explained by this study’s other findings in which the receipt of information on the use of face-masks was related to comfort of wearing the face-masks for as long as it was believed necessary as those who had received the information on the use of face-masks were more likely to be comfortable wearing the face-masks for as long as it was believed necessary. Improved knowledge is critical in shaping people’s behavior and practices particularly during disease outbreaks because knowledge is partly linked with panic emotion among most populations, which in turn influences their attitudes, perceptions, and practices as has been reported in the case of COVID-19.^74^ However, improved knowledge in the same populations may not be sufficient to cause behavioral change regarding the use of face-masks for extended durations of time. This study’s findings therefore underscore the need to bridge the gap between knowledge and practice by using more interactive and participatory training models developed in a participatory manner involving the different stakeholders, for example, through focus group discussions as well as field simulations.

In addition, efforts to train high-risk populations on the use of face-masks should be encouraged as this would ensure increased ease of using the face-masks as a protective measure against COVID-19. Also, education on other COVID-19 control measures could be disseminated as best alternatives to the adult groups who may have difficulties accepting the use of face-masks as a protective measure against COVID-19.

Regarding the finding where most of the participants had reused their face-masks, the majority having had reused them for up to a week and others for more than 1 month. Reuse was found to be associated with education status (having no formal education) and site of work (working in food markets), and this could be explained by the unavailability or shortage of the face-masks and high costs of the available face-masks in Uganda.^75,76^ Previous studies have reported the prolonged use and reuse of medical face-masks despite the recommendation for their single use because of their unavailability or shortage, especially during pandemics or extended outbreaks and other high demanding situations.^77–79^ However, the prolonged use or reuse of medical face-masks has also been documented as high-risk practices that could lead to self-contamination of the wearer and hence sources of infection.^80^

The limited supply of face-masks and the enforcement of the mandatory wearing of face-masks in all public places by the Ugandan government led to an unprecedented increase in local production of nonmedical face-masks. These masks are mostly made up of locally available materials, at both small and large scale as was reported via several local tabloids.^81^ The locally manufactured face-masks, were mostly single or double layered, and had been made out of mostly cotton fabric commonly known as “kitenge,” were cheaper and readily available to the masses. The availability of the cheap locally made face-mask could explain the finding where most of the participants owned and used nonmedical face-masks. However, similar to medical masks, the prolonged use or reuse of nonmedical face-masks could be high-risk practices that could lead to self-contamination of the wearer and hence sources of infection.^80^

This study’s findings underscore the need to sample and perform laboratory testing for both medical and nonmedical face-masks commonly circulating on the Ugandan market to assess their efficacy. This could not only help inform public health policy-makers with regard to the efficacy of the different face-masks circulating on the Ugandan market but could also inform local manufactures on ways to modify their processes so as to locally produce affordable, accessible, and safe medical and nonmedical face-masks able to offer protection, while maintaining or promoting health and also a continuous supply of the face-masks.

It is worth mentioning that higher COVID-19 knowledge, ownership, and use of face-masks and receipt of information on their use scores were found to be significantly associated with a lower likelihood of negative attitudes, perceptions, and potentially dangerous practices toward COVID-19 in this study. These findings clearly indicate the importance of improving Ugandans’ COVID-19 knowledge through health education, which may also result in improvements in their attitudes, perceptions, and practices toward COVID-19.

Our findings of the demographic factors associated with KAP toward COVID-19 and the use of face-masks are generally consistent with those of previous studies elsewhere on SARS and other viral infectious diseases.^48,53,82,83^ These findings further suggest that health education interventions would be more effective if they targeted certain demographic groups, particularly, men, the elderly, and persons with no formal education.

## CONCLUSION

Our findings suggest that Ugandan high-risk groups had good knowledge, optimistic attitudes and perceptions, and relatively appropriate practices toward COVID-19. In addition, good COVID-19 knowledge was associated with optimistic attitudes and appropriate practices toward COVID-19, suggesting that health education programs aimed at improving COVID-19 knowledge are helpful for encouraging optimistic attitudes and perceptions as well as maintaining safe practices, especially if they targeted for certain demographic groups, particularly, the men, elderly, and persons with no formal education. Furthermore, this study underscores the need for countries to adopt current guidelines provided by health agencies with regard to the use of face-masks in healthcare and community settings to prevent the transmission of COVID-19.

## References

[1] COVID-19 Dashboard, 2020 Global Web Portal. Available at: https://www.fast-trackcities.org/cities/kampala-covid. Accessed November 3, 2020.

[2] Li Q 2020 Early transmission dynamics in Wuhan, China, of novel coronavirus–infected pneumonia. N Engl J Med 382: 1199–1207. 31995857 10.1056/NEJMoa2001316 PMC7121484

[3] Cheng VCC 2020 The role of community-wide wearing of face mask for control of coronavirus disease 2019 (COVID-19) epidemic due to SARS-CoV-2. J Infect 81: 107–114. 32335167 10.1016/j.jinf.2020.04.024 PMC7177146

[4] Guan W 2020 Clinical characteristics of coronavirus disease 2019 in China. N Engl J Med 382: 1708–1720. 32109013 10.1056/NEJMoa2002032 PMC7092819

[5] Liu J Liao X Qian S Yuan J Wang F Liu Y Wang Z Wang FS Liu L Zhang Z, 2020 Community transmission of severe acute respiratory syndrome coronavirus 2, Shenzhen, China, 2020. Emerg Infect Dis 26: 1320–1323. 32125269 10.3201/eid2606.200239 PMC7258448

[6] Burke RM 2020 Active monitoring of persons exposed to patients with confirmed COVID-19—United States, January–February 2020. MMWR Morb Mortal Wkly Rep 69: 245–246. 32134909 10.15585/mmwr.mm6909e1 PMC7367094

[7] Ong SWX Tan YK Chia PY Lee TH Ng OT Wong MSY Marimuthu K, 2020 Air, surface environmental, and personal protective equipment contamination by severe acute respiratory syndrome coronavirus 2 (SARS-CoV-2) from a symptomatic patient. JAMA 323: 1610–1612. 32129805 10.1001/jama.2020.3227 PMC7057172

[8] Wu Z McGoogan JM, 2020 Asymptomatic and pre-symptomatic COVID-19 in China. Infect Dis Poverty 9: 72. 32564770 10.1186/s40249-020-00679-2 PMC7306411

[9] Wu ZY, 2020 Asymptomatic and pre-symptomatic cases of COVID-19 contribution to spreading the epidemic and need for targeted control strategies. Zhonghua Liu Xing Bing Xue Za Zhi 41: 801–805. 32274917 10.3760/cma.j.cn112338-20200406-00517

[10] Guo ZD 2020 Aerosol and surface distribution of severe acute respiratory syndrome coronavirus 2 in hospital wards, Wuhan, China, 2020. Emerg Infect Dis 26: 1583–1591. 32275497 10.3201/eid2607.200885 PMC7323510

[11] Chia PY 2020 Detection of air and surface contamination by SARS-CoV-2 in hospital rooms of infected patients. Nat Commun 11: 2800. 32472043 10.1038/s41467-020-16670-2 PMC7260225

[12] Santarpia JL 2020 Aerosol and surface contamination of SARS-CoV-2 observed in quarantine and isolation care. Sci Rep 10: 12732 Available at: 10.1038/s41598-020-69286-3. 32728118 PMC7391640

[13] Sanders JM Monogue ML Jodlowski TZ Cutrell JB, 2020 Pharmacologic treatments for coronavirus disease 2019 (COVID-19): a review. JAMA 323: 1824–1836. 32282022 10.1001/jama.2020.6019

[14] Gautret P 2020 Hydroxychloroquine and azithromycin as a treatment of COVID-19: results of an open-label non-randomized clinical trial. Int J Antimicrob Agents 10: 105949. 10.1016/j.ijantimicag.2020.105949 PMC7102549 32205204

[15] Aledort JE Lurie N Wasserman J Bozzette SA, 2007 Non-pharmaceutical public health interventions for pandemic influenza: an evaluation of the evidence base. BMC Public Health 7: 208. 17697389 10.1186/1471-2458-7-208 PMC2040158

[16] Pan A 2020 Association of public health interventions with the epidemiology of the COVID-19 outbreak in Wuhan, China. JAMA 323: 1915–1923. 32275295 10.1001/jama.2020.6130 PMC7149375

[17] Ferguson N 2020 Report 9: Impact of Non-Pharmaceutical Interventions (NPIs) to Reduce COVID-19 Mortality and Healthcare Demand. London, UK: Imperial College, 77482. 10.1007/s11538-020-00726-x PMC7140590 32270376

[18] Keni R Alexander A Nayak PG Mudgal J Nandakumar K, 2020 COVID-19: emergence, spread, possible treatments, and global burden. Front Public Health 8: 216. 32574299 10.3389/fpubh.2020.00216 PMC7270802

[19] Marasinghe KM, 2020 A systematic review investigating the effectiveness of face mask use in limiting the spread of COVID-19 among medically not diagnosed individuals: shedding light on current recommendations provided to individuals not medically diagnosed with COVID-19. 10.21203/rs.3.rs-16701/v1.

[20] Ebrahim SH Ahmed QA Gozzer E Schlagenhauf P Memish ZA, 2020 COVID-19 and community mitigation strategies in a pandemic. BMJ 368: m1066. 32184233 10.1136/bmj.m1066

[21] Cheng VCC Lau SKP Woo PCY Yuen KY, 2007 Severe acute respiratory syndrome coronavirus as an agent of emerging and reemerging infection. Clin Microbiol Rev 20: 660–694. 17934078 10.1128/CMR.00023-07 PMC2176051

[22] Cheng VCC To KKW Tse H Hung IFN Yuen KY, 2012 Two years after pandemic influenza A/2009/H1N1: what have we learned? Clin Microbiol Rev 25: 223–263. 22491771 10.1128/CMR.05012-11 PMC3346300

[23] Dixon MG Schafer IJ, 2014 Ebola viral disease outbreak—west Africa, 2014. MMWR Morb Mortal Wkly Rep 63: 548–551. 24964881 PMC5779383

[24] Pandey A Atkins KE Medlock J Wenzel N Townsend JP Childs JE Nyenswah TG Ndeffo-Mbah ML Galvani AP, 2014 Strategies for containing Ebola in west Africa. Science 346: 991–995. 25414312 10.1126/science.1260612 PMC4316831

[25] Mbonye AK Wamala JF Nanyunja M Opio A Makumbi I Aceng JR, 2014 Ebola viral hemorrhagic disease outbreak in west Africa-lessons from Uganda. Afr Health Sci 14: 495–501. 25352864 10.4314/ahs.v14i3.1 PMC4209631

[26] World Health Organization, 2020 *Advice on the Use of Masks in the Community, during Home Care and in Health Care Settings in the Context of the Novel Coronavirus (2019-nCoV) Outbreak*. Available at: https://aslm.org/wp-content/uploads/2020/05/1589476993-WHO-2019-nCov-IPC_Masks-2020.3-eng-00000004.pdf. Accessed August 5, 2020.

[27] Centers for Diseases Control, 2020 *COVID-19: Considerations for Wearing Masks*. Available at: https://www.cdc.gov/coronavirus/2019-ncov/prevent-getting-sick/cloth-face-cover-guidance.html. Accessed August 6, 2020.

[28] Chen X Ran L Liu Q Hu Q Du X Tan X, 2020 Hand hygiene, mask-wearing behaviors and its associated factors during the COVID-19 epidemic: a cross-sectional study among primary school students in Wuhan, China. Int J Environ Res Public Health 17: 2893. 10.3390/ijerph17082893 PMC7215913 32331344

[29] Freeman MC 2014 Systematic review: hygiene and health: systematic review of handwashing practices worldwide and update of health effects. Trop Med Int Health 19: 906–916. 24889816 10.1111/tmi.12339

[30] Luby SP Halder AK Huda T Unicomb L Johnston RB, 2011 The effect of handwashing at recommended times with water alone and with soap on child diarrhea in rural Bangladesh: an observational study. PLoS Med 8: e1001052. 21738452 10.1371/journal.pmed.1001052 PMC3125291

[31] Middleton JD Lopes H, 2020 Face masks in the COVID-19 crisis: caveats, limits, and priorities. BMJ 369: m2030. 32457028 10.1136/bmj.m2030

[32] MacIntyre CR Chughtai AA, 2015 Facemasks for the prevention of infection in healthcare and community settings. BMJ 350: h694. 25858901 10.1136/bmj.h694

[33] Eikenberry SE Mancuso M Iboi E Phan T Eikenberry K Kuang Y Kostelich E Gumel AB, 2020 To mask or not to mask: modeling the potential for face mask use by the general public to curtail the COVID-19 pandemic. Infect Dis Model 5: 293–308. 32355904 10.1016/j.idm.2020.04.001 PMC7186508

[34] Javid B Weekes MP Matheson NJ, 2020 COVID-19: should the public wear face masks? BMJ 369: m1442. 32273278 10.1136/bmj.m1442

[35] Brainard JS Jones N Lake I Hooper L Hunter P, 2020 Facemasks and similar barriers to prevent respiratory illness such as COVID-19: a rapid systematic review. medRxiv. 10.1101/2020.04.01.20049528. PMC7730486 33303066

[36] Klompas M Morris CA Sinclair J Pearson M Shenoy ES, 2020 Universal masking in hospitals in the COVID-19 era. N Engl J Med 382: e63. 32237672 10.1056/NEJMp2006372

[37] Uganda Ministry of Health, 2020 *Guidelines on Mask uUe*. Available at: https://www.health.go.ug/covid/wp-content/uploads/2020/05/FINAL-GUIDELINESON-MASK-USE-1-1.pdf. Accessed August 5, 2020.

[38] Steadfast Safaris, 2020 *Local Markets in Kampala, Uganda*. Available at: https://steadfastsafaris.com/uganda-safari-news/local-markets-in-kampala/. Accessed August 7, 2020.

[39] Usman IM 2020 Community drivers affecting adherence to WHO guidelines against COVID-19 amongst rural Ugandan market vendors. Front Public Health 8: 340. 32733839 10.3389/fpubh.2020.00340 PMC7357280

[40] Garda, 2020 UgandaLlockdown-Measures-to-be-Eased. Available at: https://www.garda.com/crisis24/news-alerts/343631/uganda-lockdown-measures-to-be-eased-from-june-2-update-7. Accessed November 11, 2020.

[41] Eccles R, 2002 An explanation for the seasonality of acute upper respiratory tract viral infections. Acta Otolaryngol 122: 183–191. 11936911 10.1080/00016480252814207

[42] Pica N Bouvier NM, 2012 Environmental factors affecting the transmission of respiratory viruses. Curr Opin Virol 2: 90–95. 22440971 10.1016/j.coviro.2011.12.003 PMC3311988

[43] Erfani A Shahriarirad R Ranjbar K Mirahmadizadeh A Moghadami M, 2020 Knowledge, attitude and practice toward the novel coronavirus (COVID-19) outbreak: A population-based survey in Iran. Bull World Health Organ (E-pub ahead or print 2020 Mar 30).

[44] Guyana Ministry of Public Health, 2020 Partnership with UNICEF - KAP Survey on COVID 19-Response-2020. Available at: https://www.AU15 surveymonkey.com/r/KAP_GY.AJ.

[45] Ministry of Health-Uganda, 2020 Guidelines on Preventive Measures against Corona Virus. Available at: https://www.health.go.ug/covid/document/circular-letter-no-3-of-2020-guidelineson-preventive-measures-against-corona-virus-covid-19/. Accessed August 5, 2020.

[46] Ministry of Health-Uganda, 2020 Update on Uganda’s Preparedness Situation Following the Corona Virus 2019 Outbreak in China. Available at: https://www.health.go.ug/covid/document/update-on-ugandas-preparednesssituation-following-the-corona-virus-2019-outbreak-in-china/. Accessed August 5, 2020.

[47] Ministry of Health-Uganda, 2020 Presidential Address on Corona Virus. Available at: https://www.health.go.ug/covid/document/presidential-address-on-corona-virus. Accessed August 5, 2020.

[48] Zhong BL Luo W Li HM Zhang QQ Liu XG Li WT Li Y, 2020 Knowledge, attitudes, and practices towards COVID-19 among Chinese residents during the rapid rise period of the COVID-19 outbreak: a quick online cross-sectional survey. Int J Biol Sci 16: 1745–1752. 32226294 10.7150/ijbs.45221 PMC7098034

[49] Ministry of Health-Uganda, 2020 Uganda’s Covid-19 Infections Enter Stage Three. Available at: https://ugandaradionetwork.net/story/ugandas-covid-19-infectionsenter-stage-three. Accessed August 5, 2020.

[50] Azlan AA Hamzah MR Sern TJ Ayub SH Mohamad E, 2020 Public knowledge, attitudes and practices towards COVID-19: a cross-sectional study in Malaysia. PLoS One 15: e0233668. 32437434 10.1371/journal.pone.0233668 PMC7241824

[51] Huynh G Nguyen TNH Vo KN Pham LA, 2020 Knowledge and attitude toward COVID-19 among healthcare workers at district 2 hospital, Ho Chi Minh City. Asian Pac J Trop Med 13: 260.

[52] Reuben RC Danladi MMA Saleh DA Ejembi PE, 2020 Knowledge, attitudes and practices towards COVID-19: an epidemiological survey in north-central Nigeria. J Community Health 1–14. Available at: 10.1007/s10900-020-00881-1. 32638198 PMC7338341

[53] Olum R 2020 Perspective of medical students on the COVID-19 pandemic: survey of nine medical schools in Uganda. JMIR Public Health Surveill 6: e19847. 32530815 10.2196/19847 PMC7307324

[54] Ministry of Health-Uganda_Offcial Website, 2020 Available at: https://www.health.go.ug/.

[55] Makerere University-COVID-19 Resource Center, 2020 Available at: https://coronavirus.mak.ac.ug/.

[56] Borchert M 2011 Ebola haemorrhagic fever outbreak in Masindi district, Uganda: outbreak description and lessons learned. BMC Infect Dis 11: 357. 22204600 10.1186/1471-2334-11-357 PMC3276451

[57] MacNeil A 2011 Filovirus outbreak detection and surveillance: lessons from Bundibugyo. J Infect Dis 204 (Suppl 3): S761–S767. 21987748 10.1093/infdis/jir294

[58] Nabukenya I 2014 Is Uganda a hub for zoonotic disease outbreaks? Lessons and challenges from Ebola, Marburg, Yellow fever and anthrax outbreaks. Int J Infect Dis 21: 238.

[59] H.E. President Yoweri Museveni Addresses the Nation on the Coronavirus Epidemic, 2020 Available at: http://statehouse.go.ug/sites/default/files/files/presidential-statements/address-coronaAU18virus-4-may-2020-converted.pdf. Accessed August 5, 2020.

[60] Feng S Shen C Xia N Song W Fan M Cowling BJ, 2020 Rational use of face masks in the COVID-19 pandemic. Lancet Respir Med 8: 434–436. 32203710 10.1016/S2213-2600(20)30134-X PMC7118603

[61] Lau LL Hung N Go DJ Ferma J Choi M Dodd W Wei X, 2020 Knowledge, attitudes and practices of COVID-19 among income-poor households in the Philippines: a cross-sectional study. J Glob Health 10: 011007. 32566169 10.7189/jogh.10.011007 PMC7294392

[62] Haffajee RL Mello MM, 2020 Thinking globally, acting locally—the US response to COVID-19. N Engl J Med 382: e75. 32240580 10.1056/NEJMp2006740

[63] Saqlain M Munir MM Rehman SU Gulzar A Naz S Ahmed Z Tahir AH Mashhood M, 2020 Knowledge, attitude, practice and perceived barriers among healthcare professionals regarding COVID-19: a cross-sectional survey from Pakistan. J Hosp Infect 105: 419–423. 32437822 10.1016/j.jhin.2020.05.007 PMC7211584

[64] Kasusse M, 2005 Bridging the digital divide in sub-Saharan Africa: the rural challenge in Uganda. Int Inf Libr Rev 37: 147–158.

[65] Lehmann U Friedman I Sanders D, 2004 Review of the Utilisation and Effectiveness of Community-based Health Workers in Africa. Global Health Trust, Joint Learning Initiative on Human Resources for Health and Development (JLI), JLI Working Paper, pp. 4–1.

[66] Duell N 2018 Age patterns in risk taking across the world. J Youth Adolesc 47: 1052–1072. 29047004 10.1007/s10964-017-0752-y PMC5878702

[67] Liang W Shediac-Rizkallah MC Celentano DD Rohde C, 1999 A population-based study of age and gender differences in patterns of health-related behaviors. Am J Prev Med 17: 8–17. 10429747 10.1016/s0749-3797(99)00040-9

[68] Byrnes JP Miller DC Schafer WD, 1999 Gender differences in risk taking: a meta-analysis. Psychol Bull 125: 367–383.

[69] Novak JR Peak T Gast J Arnell M, 2019 Associations between masculine norms and health-care utilization in highly religious, heterosexual men. Am J Mens Health 13: 1557988319856739. 31184245 10.1177/1557988319856739 PMC6560804

[70] Byanaku A Ibrahim M, 2020 Knowledge, attitudes, and practices (KAP) towards COVID-19: a quick online cross-sectional survey among Tanzanian residents. medRxiv. 10.1101/2020.04.26.20080820.

[71] Ng K Poon BH Kiat Puar TH Shan Quah JL Loh WJ Wong YJ Tan TY Raghuram J, 2020 COVID-19 and the risk to health care workers: a case report. Ann Intern Med 172: 766–767. 32176257 10.7326/L20-0175 PMC7081171

[72] Bartoszko JJ Farooqi MAM Alhazzani W Loeb M, 2020 Medical masks vs N95 respirators for preventing COVID‐19 in healthcare workers: a systematic review and meta‐analysis of randomized trials. Influenza Other Respi Viruses 14: 365–373. 10.1111/irv.12745 PMC7298295 32246890

[73] Ferdousa MZ Islama MS Sikdera MT Md AS, 2020 Knowledge, attitude, and practice regarding COVID-19 outbreak in Bangladesh: an online-based cross-sectional study. PLoS One 14: 365. 10.1371/journal.pone.0239254 PMC7546509 33035219

[74] Abdulkareem SA Augustijn EW Filatova T Musial K Mustafa YT, 2020 Risk perception and behavioral change during epidemics: comparing models of individual and collective learning. PLoS One 15: e0226483. 31905206 10.1371/journal.pone.0226483 PMC6944362

[75] Chan KH Yuen KY, 2020 COVID-19 epidemic: disentangling the re-emerging controversy about medical facemasks from an epidemiological perspective. Int J Epidemiol 49: 1063–1066. 32232402 10.1093/ije/dyaa044 PMC7184463

[76] Siow WT Liew MF Shrestha BR Muchtar F See KC, 2020 Managing COVID-19 in resource-limited settings: critical care considerations. Crit Care 24: 167. 32321566 10.1186/s13054-020-02890-x PMC7175447

[77] CDC, 2020 Strategies for Optimizing the Supply of N95 Respirators: Crisis/Alternate Strategies. Available at: https://www.cdc.gov/coronavirus/2019-ncov/hcp/respirators-strategy/crisis-alternate-strategies.html. Accessed November 30, 2020.

[78] Michaels D Wagner GR, 2020 Occupational Safety and Health Administration (OSHA) and worker safety during the COVID-19 pandemic. JAMA 324: 1389–1390. 10.1001/jama.2020.16343 32936212

[79] Chughtai 2020 Policies on the use of respiratory protection for hospital health workers to protect from coronavirus disease (COVID-19). Int J Nurs Stud 105: 103567. 32203757 10.1016/j.ijnurstu.2020.103567 PMC7174826

[80] Chughtai AA Seale H Islam MS Owais M Macintyre CR, 2020 Policies on the use of respiratory protection for hospital health workers to protect from coronavirus disease (COVID-19). Int J Nurs Stud 105: 103567. 32203757 10.1016/j.ijnurstu.2020.103567 PMC7174826

[81] UNBS Certifies Six Companies to Manufacture Face Masks, 2020 Available at: https://www.independent.co.ug/unbs-certifiessix-companies-to-manufacture-face-masks. Accessed August 8, 2020.

[82] Zhang X Sun Y Ye D Sun Z Su H Ni J, 2003 Analysis on mental health status of community residents in Hefei during SARS spread. China J Dis Contr Prev 7: 280–282.

[83] Jiao J Tang X Li H Chen J Xiao Y Li A, 2005 Survey of knowledge of villagers in prevention and control of SARS in Hainan province. China Trop Med 5: 703–705.

